# Environmental Pollution, Biomarkers, and Dementia: An Evidence Review

**DOI:** 10.3390/ijms27188224

**Published:** 2026-09-15

**Authors:** Yicong Huang, Chaewon Park, Artitaya Lophatananon, Kenneth R. Muir

**Affiliations:** Division of Population Health, Health Services Research and Primary Care, School of Health Sciences, Faculty of Biology, Medicine and Health, The University of Manchester, Manchester M13 9PT, UK; yicong.huang@manchester.ac.uk (Y.H.); chaewon.park@manchester.ac.uk (C.P.); artitaya.lophatananon@manchester.ac.uk (A.L.)

**Keywords:** dementia, Alzheimer’s disease, air pollution, environmental chemical pollutants, epigenetics, genetics, metabolomics

## Abstract

With rapid global urbanization, exposure to environmental pollutants has emerged as a potentially important determinant of cognitive health. We therefore systematically searched PubMed and Embase for epidemiological studies published between 2020 and 2026 and reviewed the associations between ambient air pollution, environmental chemical pollutants, and dementia. Evidence on potential biological mechanisms was identified to support the biological plausibility of the observed associations. Epidemiological evidence indicated that long-term exposure to particulate matter (PM_2.5_), nitrogen oxides (NO_2_), and black carbon was associated with increased dementia risk, whereas evidence for PM_10_ and ozone remained inconclusive. Plastic-associated chemicals, including phthalates and bisphenols, heavy metals (Lead-Pb and Cadmium-Cd), and per- and polyfluoroalkyl substances (PFOS), were linked with poorer cognitive function and dementia-related outcomes; findings for pesticides and herbicides were inconsistent. In terms of possible mechanisms, molecular evidence suggests that air pollution and environmental chemical pollutants may promote oxidative stress, neuroinflammation, metabolic dysfunction, Alzheimer’s disease pathology, and vascular damage, although evidence for epigenetic mechanisms remains more limited for environmental chemical pollutants. Overall, the evidence remains limited due to the predominance of observational studies. Future research that integrates molecular biomarkers and causal inference techniques is needed to better understand the role of environmental pollutants in dementia.

## 1. Introduction

With rapid global urbanization, environmental exposures have become an increasingly important determinant of human health. Urbanization has substantially altered the human exposome by increasing exposure to a wide range of environmental pollutants through transportation, industrial activities, and the widespread use of consumer products. Ambient air pollution and environmental chemical pollutants are of particular interest because of their widespread exposure and potential contribution to neurodegenerative diseases. Because exposure to these pollutants is potentially modifiable through environmental regulation, industrial policy, and public health intervention, clarifying their contribution to dementia risk may inform population-level prevention strategies and policy action to reduce the future burden of dementia.

The World Health Organization identifies five major ambient air pollutants: particulate matter (PM), carbon monoxide (CO), ozone (O_3_), nitrogen dioxide (NO_2_), and sulfur dioxide (SO_2_); these pollutants arise from sources, including fossil fuel combustion, transport, and industrial processes [1]. Ambient air pollution is one of the most extensively studied environmental risk factors for dementia. Notably, 91% of urban residents live in areas that fail to meet World Health Organisation (WHO) global air quality guidelines [2], and autopsy samples from children and young adults in highly polluted areas showed extracellular deposition of amyloid-beta (Aβ) and phosphorylated tau (pTau), which are key hallmarks of Alzheimer’s disease [3]. In addition, nanosized particulate matter (nPM) deposits have been discovered in the frontal cortex of human brains [4]. Moreover, the 2024 update of the Lancet Commission on Alzheimer’s and related dementias identified air pollution as one of the fourteen potentially modifiable risk factors of dementia [5]. A systematic review conducted in 2019 found that greater exposure to air pollutants, including fine particulate matter (PM_2.5_), NO_2_/nitrogen oxides (NO_x_), and CO, has been associated with an increased risk of dementia [6].

Endocrine-disrupting chemicals (EDCs) are exogenous substances that interfere with hormonal signaling and alter endocrine system function [7]. They are now widely used in consumer and household products, including phthalates in plastics and parabens in personal care products. Evidence suggests EDCs may affect neuronal development and contribute to neurodegenerative disease [7,8]. However, not all non-air-pollution chemical exposures considered in this review are EDCs. Therefore, we use the broader term environmental chemical pollutants to refer collectively to non-air-pollution chemical exposures, including plastic-associated endocrine-disrupting chemicals, PFAS, pesticides/herbicides, persistent organic pollutants, and heavy metals. These exposure classes differ in their chemical properties and biological mechanisms and are therefore discussed separately rather than treated as a single EDC category. Heavy metals were retained because of their established environmental neurotoxicity and relevance to cognitive outcomes, but they are not assumed to act primarily through endocrine-disrupting pathways.

Although epidemiological studies increasingly support associations between environmental pollutants and dementia, most evidence remains observational and therefore cannot establish causality. Biological mechanisms and molecular biomarkers therefore provide important insights into how exposure links to disease. Pollutant gases can reach the brain by diffusing through the lung epithelial and vascular endothelial layers, or by travelling directly along the olfactory nerve. Once in the brain, these pollutants can promote oxidative stress, neuroinflammation, blood–brain barrier (BBB) disruption, and neuronal damage, ultimately contributing to neuronal injury and the accumulation of Alzheimer’s disease (AD) pathology [9]. EDC exposure is thought to act through similar mechanisms, including oxidative stress, neuroinflammation, and disruption of hormone signaling, that are critical for normal brain function, synaptic plasticity, and cognitive aging [8,10]. Molecular biomarkers provide measurable evidence of these mechanisms in humans, helping to bridge the gap between exposure and disease and strengthen causal inference. For example, lipid metabolism has been shown to mediate the association between PM_2.5_ exposure and dementia risk [11]. Similarly, higher exposure to per- and polyfluoroalkyl substances (PFAS) has been associated with elevated serum lipid levels [12]. Abnormal lipid metabolism can exacerbate neuroinflammation and accelerate neurodegenerative processes associated with dementia [13].

Previous reviews have largely considered air pollution or EDCs separately and have focused predominantly on epidemiological evidence. This review integrates recent 5-year epidemiological findings with emerging evidence from molecular biomarkers, including metabolomic, proteomic, epigenetic, and genetic studies. We aim to provide a more comprehensive mechanistic framework that links environmental exposures to neurodegeneration and dementia. By combining these complementary lines of evidence, we also highlight opportunities for biomarker-based risk stratification and future prevention strategies.

## 2. Methods

This review comprised two components: (1) a systematic search and synthesis of epidemiological evidence examining associations between environmental pollutants and dementia-related outcomes, and (2) a broader search of mechanistic evidence to identify biological pathways that may explain and support the associations observed in epidemiological studies. Thus, the epidemiological evidence provides the primary evidence for the associations of interest, whereas the mechanistic literature provides complementary evidence regarding their biological plausibility.

For the epidemiological component, a structured literature search was performed to find relevant epidemiological studies from January 2020 to March 2026, as earlier studies have already been comprehensively summarized [6,14]. Two electronic databases, PubMed and Embase, were searched using combinations of terms related to environmental pollutants and dementia-related outcomes.

The search strategy combined terms related to environmental pollutants and dementia outcomes. The following keywords and their combinations were used: ((phthalates[Title/Abstract]) OR (heavy metals[Title/Abstract]) OR (air pollution[Title/Abstract]) OR (PM2.5[Title/Abstract]) OR (PM10[Title/Abstract]) OR (NO2[Title/Abstract]) OR (pesticides[Title/Abstract]) OR (“Endocrine-disrupting chemicals”[Title/Abstract]) OR (PFAS[Title/Abstract]) OR (“per- and polyfluoroalkyl substances”[Title/Abstract])) AND (“Alzheimer Disease”[Title/Abstract] OR “cognitive impairment”[Title/Abstract] OR MCI[Title/Abstract] OR dementia[Title/Abstract] OR “mild cognitive impairment”[Title/Abstract]). The full search terms are included in the Supplementary Materials. As randomized controlled trials are not feasible for investigating the effects of environmental exposures on dementia risk, this review focused on observational evidence, including cohort, case-control, and cross-sectional studies. Studies were excluded if they were non-human, qualitative, non-peer-reviewed publications, case reports, case series, conference abstracts, study protocols, editorials, letters, or book chapters. Additionally, studies were excluded if the full text was unavailable or if they targeted a single gender.

Y.H. carried out the literature search and screening according to predefined eligibility criteria. C.P. reviewed the excluded studies to assess the appropriateness of exclusion decisions, and any disagreements were resolved through discussion. Extracted variables included first author, year, country, sample size, study design, duration, exposure, and dementia or cognitive outcomes. Longitudinal cohort studies were assessed using the Newcastle–Ottawa Quality Assessment Scale (NOS) [15], and cross-sectional studies were assessed using the adapted NOS (NOS-xs) [16]. A study with a score of 7 or more can be considered high quality, with a low risk of bias. Epidemiological findings were synthesized systematically, with emphasis on the magnitude, direction, and consistency of associations between environmental pollutants and dementia-related outcomes.

In addition, a broader literature search was conducted to identify mechanistic evidence relevant to the potential biological pathways linking environmental pollutant exposure with dementia. Mechanistic studies published between 2010 and 2025 were identified through literature searches of PubMed using combinations of the terms: air pollution, particulate matter, endocrine-disrupting chemicals, Alzheimer’s disease, dementia, biomarkers, epigenetics, proteomics, and metabolomics.

The mechanistic search was not intended to constitute a systematic review of all mechanistic studies. Its purpose was to find evidence that could support biological explanations and clarify the associations observed in epidemiological research. Human biomarker studies, post-mortem investigations, experimental models, and translational studies were included where they provided evidence supporting biological mechanisms and pathologies relevant to the association of pollutants with neurodegenerative disease risk.

Mechanistic findings were synthesized narratively, with emphasis on molecular biomarkers and pathways, including epigenetic, metabolomic, and proteomic alterations, potentially linking air pollution and environmental chemical pollutants to dementia-related outcomes. Where relevant, endocrine-disrupting mechanisms were considered specifically for chemicals with established or suspected endocrine-disrupting activity.

## 3. Epidemiological Evidence

**Figure 1:**
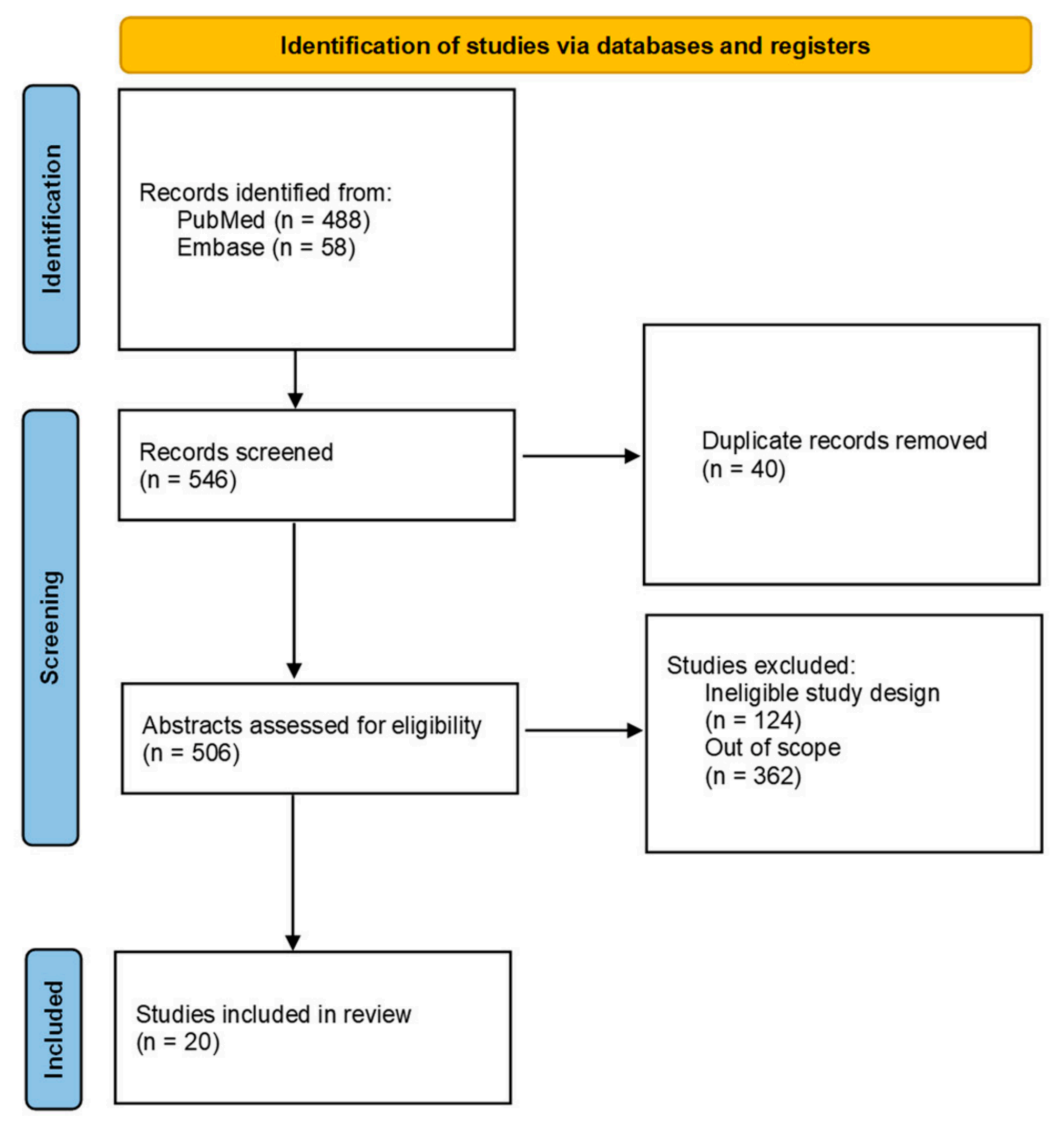
The PRISMA flow diagram of the study search and selection process.

A total of 546 publications were identified, of which 20 were included in the final epidemiological evidence (Figure 1). Nine prospective cohort studies [17,18,19,20,21,22,23,24,25] examined the relationship between exposure to ambient air pollution and dementia risk. The main air pollutants included PM_2.5_, NO_2_, PM_10_, ozone, and black carbon. Exposure was assessed using model-based approaches, including land-use regression, dispersion models, and chemical transport models (CTMs), often linked to participants’ residential histories. Across studies, exposure windows varied, ranging from single-year baseline estimates to cumulative averages over 5–20 years or life-course exposures. Effect estimates were reported per different concentration units (e.g., 1–10 µg/m^3^, per IQR, or by tertiles). This heterogeneity precluded direct comparison of effect sizes. Therefore, synthesis focuses on the direction and relative consistency of associations. Nine cross-sectional studies and two prospective cohorts examined the relationship between environmental chemical pollutant exposure and dementia risk, including heavy metals, plastic-associated chemicals, per- and polyfluoroalkyl substances (PFAS), herbicides, and pesticides [26,27,28,29,30,31,32,33,34,35,36].

Studies included populations from multiple countries, including the United States (n = 8), the United Kingdom (n = 4), China (n = 2), France (n = 2), Australia (n = 1), Denmark (n = 1), Spain (n = 1), and Sweden (n = 1). Sample sizes ranged from 65 to over 900,000. The follow-up periods of longitudinal studies ranged from 2 years to over 12 years. Table 1, Table 2 and Table 3 summarized the characteristics and main findings of studies on air pollution and EDC exposure in relation to dementia risk, respectively.

### 3.1. Air Pollution

#### 3.1.1. PM_2.5_

PM_2.5_ refers to fine particulate matter with diameters ≤2.5 μm. Across the eight studies, six reported effect estimates above the null, including four that showed statistically significant positive associations with PM_2.5_ [17,19,21,25]. These studies reported approximately 8–20% higher hazards of dementia per specified increment in PM_2.5_ exposure. Elevated dementia risk was observed even at relatively low mean PM_2.5_ concentrations (9.9 µg/m^3^) [19], suggesting possible adverse effects below current guideline thresholds. However, in settings with very low PM_2.5_ levels (5.6 µg/m^3^), the association was no longer statistically significant [20]. Source-specific analyses identified agriculture and wildfire as major contributors [25].

#### 3.1.2. NO_2_

Three of six studies found that increased NO_2_ exposure was associated with a higher hazard of dementia, with hazard ratios (HRs) ranging from 1.09 to 1.25 per study-specific exposure increment [17,19,21]. However, other studies reported inconsistent results. Jutila et al. (2024) reported a positive but non-significant association [18], while Wood et al. (2022) found no significant inverse association [22]. Wu et al. reported cohort-specific differences, with one cohort indicating a positive association and another showing null effects [24].

#### 3.1.3. Black Carbon (BC)

Black carbon (BC) is a marker of traffic-related combustion. Evidence for black carbon, although limited to two studies, was consistently positive. One analysis found a 23% higher hazard of dementia per 0.5 × 10^−5^/m increase in BC [17], while another, using PM_2.5_ absorbance as a proxy for BC exposure, reported a 3% higher hazard per 1 µg/m^3^ [19].

#### 3.1.4. PM_10_ and Ozone (O_3_)

In contrast, findings for total PM_10_ and ozone (O_3_) were generally null or inconsistent. Three studies reported HRs close to or overlapping the null, and no study found strong positive associations for total PM_10_ [20,22,24]. However, source-specific analyses in Oudin et al. (2024) suggested a positive association for traffic-related PM_10_, with a 35% higher hazard of dementia per tertile increase in exposure during the 1–5-year window, although this association was not observed for the 6–10-year window [20]. Therefore, PM_10_ composition and source may be more important determinants of dementia risk than total PM_10_ concentration. For ozone, the available evidence does not support a consistent association between ozone exposure and dementia risk. Wood et al. (2022) found a non-significant protective association [22], whereas Wu et al. (2024) found a significant inverse association in the CFAS II cohort but a non-significant positive association in the CFAS Wales cohort [24]. One recent study investigating combined PM_2.5_, PM_10_, and NO_2_ exposure found no significant association with dementia [23].

In summary, despite the heterogeneity, most evidence supports positive associations for PM_2.5_, NO_2_, and black carbon, whereas associations with PM_10_ and ozone were mixed or null. Several studies also identified individual vulnerability and timing of exposure as important modifiers of dementia risk. Older adults (≥75 years), socioeconomically disadvantaged populations, and individuals with stroke or cardiometabolic disease had an increased dementia risk when exposed to air pollution [17]. Evidence that genetic susceptibility modifies the association between air pollution and dementia is inconsistent. Ma et al. identified significant additive interactions between air pollution and both *APOE *ε4 status and polygenic risk score [19], whereas Semmens et al. found no multiplicative interaction with *APOE *ε4 and suggested that this may reflect a stronger association of air pollution with the vascular dementia (VaD) pathway than Alzheimer’s disease (AD) [21]. A life-course analysis found that in utero and early-life exposures to PM_2.5_ and NO_2_ significantly increased dementia risk, supporting the neurotoxic potential of early and cumulative exposure [18].

### 3.2. Environmental Chemical Pollutants

#### 3.2.1. Heavy Metals

Four cross-sectional studies investigated associations between heavy metal exposure in blood or urine and cognitive function or neurological biomarkers, such as serum neurofilament light chain (sNfL) [26,32,33,34]. Heavy metals showed a significant and consistent association between environmental exposure and cognitive decline, with specific metals acting as primary neurotoxicants while others serve protective roles. Higher levels of lead (Pb), cadmium (Cd), copper (Cu), and arsenic (As) are generally associated with poorer cognitive function and an increased risk of mild cognitive impairment. Of these metals, Cd and Pb emerged as the most consistently implicated toxic metals [26,32,33,34]. In contrast, selenium (Se) and zinc (Zn) were found to have protective effects on cognitive function, which consistently appear to attenuate or counteract the adverse effects of neurotoxic metals [26,33,34]. This pattern is further supported by evidence that higher Cd and Pb concentrations were associated with increased serum neurofilament light chain (sNfL), a marker of neuronal injury, whereas Se was associated with lower sNfL levels, suggesting a potential protective effect against heavy metal-induced neuronal damage [26].

#### 3.2.2. Plastic-Associated Chemicals

Two cross-sectional studies assessed plastic-associated chemicals, including phthalates (PAEs), phenols, parabens, and bisphenols, using urinary metabolite concentrations, and examined their associations with cognitive function, Alzheimer’s disease biomarkers (PET-Aβ status), and clinical status (MCI/AD). Both studies reported that mixed exposure to phthalates and bisphenols was associated with poorer cognitive performance or clinical status [28,29]. In a study of 856 older adults in the United States, higher urinary concentrations of phenol, paraben, and phthalate metabolites were significantly associated with poorer cognitive performance, particularly among men. Mono(2-ethylhexyl) phthalate (MEHP) was identified as the main contributor to lower Immediate Recall Test scores [28]. In another study of 191 older adults in Australia, mixtures of bisphenols and phthalates were significantly associated with MCI/AD status. Higher bisphenol S (BPS) concentrations were associated with amyloid-β positivity (Aβ+), whereas unexpectedly lower MEHP concentrations were also associated with Aβ+; this latter finding should therefore be interpreted cautiously [29]. Overall, these findings suggest that mixed exposure to plastic-associated chemicals may contribute to cognitive health, although the effects of individual chemicals remain inconsistent.

#### 3.2.3. Per- and Polyfluoroalkyl Substances (PFAS)

Per- and polyfluoroalkyl substances (PFASs) are a large group of man-made chemicals, known as “forever chemicals” because of their highly persistent nature in the environment and living organisms, leading to their accumulation over time. A prospective cohort study identified a signature of nine dietary contaminants, including four PFASs, that was associated with a significantly higher hazard of incident dementia among older adults consuming a high-fat diet (>35% of energy intake from fat). In single-contaminant analyses, perfluorooctanesulfonic acid (PFOS) showed the strongest association with incident dementia (HR = 1.43) [35]. Consistent with these findings, a pilot study of 65 firefighters also found that higher PFAS mixture exposure was associated with poorer processing speed, with PFOS identified as the primary contributor to the observed association (effect estimate [β], 95%CI: −0.58, −0.91–−0.24) [36]. These studies indicate a potential link between PFAS exposure and impaired cognitive outcomes, and PFOS has repeatedly been identified as the major contributor among individual PFAS compounds.

#### 3.2.4. Pesticides and Herbicides

A large-scale cross-sectional study in Spain found that regions with high pesticide exposure had significantly higher AD prevalence and a greater likelihood of individuals developing AD than low-usage areas (adjusted OR (95%CI) = 1.64 (1.41–1.89)). This risk appeared particularly pronounced for women (adjusted OR (95%CI) = 1.17 (1.15–1.80)) [31]. Another cross-sectional study identified a U-shaped relationship between urinary concentrations of the common herbicide 2,4-dichlorophenoxyacetic acid (2,4-D) and cognitive impairment among older U.S. adults, especially in men. Moderate urinary levels (0.315–0.566 μg/L) were associated with the lowest odds of cognitive impairment, whereas the odds increased at both lower and higher exposure levels [30]. Conversely, a prospective cohort study found that higher plasma concentrations of p,p’-DDT were associated with lower dementia risk; however, this inverse association has limited biological plausibility and should be interpreted with caution [27].

Taken together, current evidence suggests that mixed environmental chemical pollutant exposure may contribute to cognitive decline and dementia. Among individual chemicals, Cd, Pb, BPS, and PFOS showed the most consistent evidence of neurotoxicity, whereas associations for several other compounds remained inconsistent across studies.

### 3.3. Quality Assessment

The methodological quality of the included studies was generally high, with all studies considered to have a low risk of bias. Among the eleven cohort studies, three studies scored 7/9 [22,23,24], two scored 8/9 [18,35], and six scored 9/9 on the NOS [17,19,20,21,25,27] (Table S1). Among the nine cross-sectional studies, one study scored 7/9 [36], two scored 8/9 [29,31], and six scored 9/9 [26,28,30,32,33,34] on the NOS-xs (Table S2). The main potential source of bias across the cohort studies was related to outcome assessment and follow-up. Four studies had relatively high attrition during follow-up, which may have introduced selection bias if participants who remained in the study differed systematically from those lost to follow-up [18,22,23,24]. Two studies had a relatively short follow-up period of only two years, potentially limiting their ability to capture the longer-term effects of environmental exposures on dementia outcomes [23,24]. In addition, one study relied on self-reported or proxy-reported physician diagnoses of dementia rather than record linkage or independent outcome assessment [22]. The risk of bias in cross-sectional studies was mainly due to the absence of reported non-response rates. Additionally, two studies had limitations in the representativeness of their study populations, which may restrict the generalizability of their findings. One study was conducted exclusively among firefighters [36], while another analyzed a selected sub-cohort from a larger parent cohort [29].

Although all included studies were classified as having a low risk of bias based on their NOS or NOS-xs scores, limitations inherent to observational study designs should be acknowledged. Both longitudinal cohort and cross-sectional studies may be affected by reverse causality and residual confounding. Cross-sectional studies have greater limitations because exposure is often assessed using blood or urine concentrations measured at a single time point, which may not accurately reflect long-term exposure because of temporal variability and measurement error. Therefore, these findings only support an association and do not provide sufficient evidence to establish causality.

#### 3.3.1. Evidence of Molecular Biomarkers

##### Oxidative Stress and Neuroinflammation

Emerging evidence suggests that exposure to air pollutants contributes to neurodegeneration through biological mechanisms involving oxidative stress, neuroinflammation, and amyloid pathology (Table 4). Studies in post-mortem brain tissues and cellular models have demonstrated that higher exposure to NO_2_ and PM_2.5_ is associated with higher centiloid values, indicating greater Aβ accumulation [3,37].

Experimental studies showed increased pro-amyloidogenic processing of the amyloid precursor protein (*APP*) from nanosized PM (nPM) exposure in neuroblastoma cells, suggesting corresponding impacts of PM_2.5_ exposure on increasing the production of reactive oxygen species (ROS) [37,38]. This is supported by a study on microglial cells, where high-dose PM_2.5_ exposure decreased cell survival as a result of neuroinflammation and the production of ROS, leading to neuronal injury and apoptosis [37].

Consistent with these findings, positive associations have been reported between PM_2.5_ and PM_10_ exposure and cerebrospinal fluid (CSF) levels of neurofilament light chain (NfL), a marker of neuronal injury [3]. Collectively, these findings support a potential pathway whereby air-pollution-induced oxidative stress and neuroinflammation contribute to neuronal damage and impaired protein clearance.

##### Metabolic Dysregulation

Metabolic profiling showed that exposure to PM is associated with disruptions in lipid, fatty acid, amino acid, and energy metabolism. A positive relationship was identified between PM_10_ and PM_2.5_ and linoleic acid to total fatty acid percentage (linoleic acid%), lactate, and triglycerides in large low-density lipoprotein (L-LDL-TG). Meanwhile, the reverse coefficient of the metabolic signatures was phospholipids to total lipids in small LDL percentage (S-LDL-PL%) and cholesterol in medium VLDL (M-VLDL-C) [39]. Relatedly, PM_2.5_ has a potential negative association with diglycerides, which are converted to triglycerides [40]. Such alterations may be relevant to cognitive decline, as reduced triglycerides are known to be reflected in dementia patients or the elderly with faster cognitive decline [41].

Alterations in redox-related metabolites provide further evidence of metabolic disturbance. A cohort-based study showed a negative association between cysteinylglycine (cys-gly) and exposure to PM_2.5_ [40]. Cys-gly is an amino acid derivative produced when reduced glutathione (GSH) undergoes degradation catalysis, therefore acting as an indicator of GSH levels. The negative association between cys-gly levels and PM_2.5_ exposure implies reduced GSH and can be interpreted as the disturbed maintenance of redox homeostasis and cellular stress responses [40,42]. Together, these metabolomic findings suggest that air pollution may influence neurodegenerative risk not only through direct oxidative and inflammatory processes but also through broader metabolic dysregulation [38,39].

##### Epigenetic and Genetic Alteration

Low-dose, combined exposure to PM_2.5_ and SO_2_ demonstrated that coal-burning sites increased tau and pTau levels in cultured primary cortical neurons, possibly mediated by reduced levels of human miRNA, which targets the microtubule-associated protein Tau (*MAPT*) gene involved in tau protein regulation [43]. The potential mediating effect of DNA methylation on the association between PM_2.5_ exposure and the neuropathological alterations of AD was addressed by Li et al., who identified 24 CpG sites in the prefrontal cortex and showed the correlation between PM_2.5_ exposure and neuropathology markers. Differentially methylated cg25433380 and cg10495669 were found to be sites assigned to the *RBCK1* gene, which regulates the activation of the inflammatory response mediator, nuclear factor kappa B (NF-κB), which is induced by proinflammatory cytokines [44]. In addition, hypermethylation at CpG2 and CpG7 sites in peripheral brain-derived neurotrophic factor (*BDNF*) promoters was interrelated with PM above thresholds and long-term NO_2_ exposure, providing further evidence on the relationship between brain-related epigenetic changes and environmental pollutants [45]. Studies found that histone modifications cause lower nuclear levels of the inactive chromatin markers H3K9me2 and 3, along with the DNA double-strand break marker γ-H2AX in young adults from highly polluted areas, which aligned with pTau and Aβ plaques [46,47].

Epigenetic alterations have also been observed in AD pathology. Hypermethylation patterns in the hippocampus and gene-specific alterations in the cerebral cortex have been reported in patients with AD, including reduced DNA methylation within the *APP* promoter region accompanied by increased Aβ accumulation [47,48]. These pathological variations are controlled by key epigenetic mechanisms, including non-coding RNAs (ncRNAs), DNA methylation, and histone modification, comprising mitotically stable, potentially reversible changes in gene expression without altering the DNA sequence. Chronic exposure to stressors and toxins is addressed as a factor that triggers epigenetic changes and impacts the brain, including neuronal and glial cell differentiation, modification of synaptic plasticity, neurotransmission, and neuroinflammation, possibly promoting neuropathological outcomes [4,40]. NO_2_ exposure above the threshold increased the risk of inhibiting neural plasticity, depression, cognitive decline, and the development of AD [43]. Histone modifications aligning with pTau and Aβ plaques, caused by chronic exposure to pollutants, indicated a potential relationship between chromatin silencing caused by environmental pollutants and DNA damage [46,47]. Although evidence specifically implicating epigenetic mechanisms in dementia remains limited, findings to date provide preliminary evidence for the molecular effects of environmental pollutants.

##### Molecular Pathways Associated with Environmental Chemical Pollutants

Endocrine-disrupting chemicals (EDCs) are exogenous compounds that interfere with endocrine function and hormone signaling. Among the environmental chemical pollutants considered in this review, plastic-associated chemicals such as bisphenols and phthalates represent important classes with endocrine-disrupting activity. Emerging evidence suggests that EDC exposure may influence molecular pathways relevant to neurodegeneration. One study identified 27 EDC-responsive genes potentially associated with AD pathogenesis, including genes involved in immune regulation and mitochondrial function [49]. In addition, evidence relating to phthalates and bisphenol S (BPS) suggests that these exposures may disrupt metabolic pathways relevant to neurological disease, including glucose and thiamine metabolism, phosphatidylserine synthesis, and glutathione (GSH) synthesis [42].

**Table 4:** Summary of studies investigating molecular biomarkers and multi-omics alterations associated with environmental pollutant exposure and dementia.

Author (Year)	Study Design	Category of Exposure/Measurement	Biomarkers	Disease	Sample Size	Outcomes
Alemany et al. (2021, Spain) [3]	Nested longitudinal study (of ALFA cohort)	NO_2_, PM_2.5_, PM_2.5_ absorbance, and PM_10_ (using land use regression model—2009)	CSF levels of Aβ42, Aβ40, p-Tau, t-Tau, Nfl, and centiloid	AD	156	– greater exposure to NO_2_ and PM_2.5_ absorbance = ↑ brain Aβ deposition level (stronger in *APOE*-ɛ4) – greater exposure to PM_2.5_ and PM_10_ = ↑ CSF Nfl level
Tian et al. (2024, UK) [39]	Prospective cohort study (UK Biobank cohort study)	NO_2_, PM_2.5_, PM_10_, and NO_x_ (annual concentrations of air pollution were assessed)	251 metabolomic biomarkers (170 absolute metabolites and 81 composite ratios spanning lipoprotein lipids, fatty acids, and low-molecular-weight metabolites)	Dementia	192,300 dementia-free participants	– metabolites from lipoprotein subclasses played essential mediating roles – stronger association for male *APOE*-ɛ4 non-carriers
Kalia et al. (2023, US) [40]	Community-based cohort (1995–2015)	NO_2_, PM_2.5_, and PM_10_	79 features associated with PM2.5 exposure (amino acid metabolism, energy production, and oxidative stress response)	Dementia	107	– PM_2.5_ exposure may alter metabolic outcomes in older population
Ku et al. (2017, China) [43]	In vitro experimental study	PM_2.5_ and SO_2_	MicroRNAs (miR-337-5p)	Neurodegenerative disorders	69	– PM_2.5_, SO_2_ co-exposure = neuronal apoptosis, ↓ PSD-95, ↓ NMDA-NR2B, and ↑ tau phosphorylation
Li et al. (2024, USA) [44]	Cross-sectional analysis	Traffic-related PM_2.5_ (air quality models)	Aβ plaques, Nfl, and 24 CpG sites that are neuropathology markers (cg25433380, cg10495669, and *RBCK1* gene)	AD	159	– DNAm related to neuroinflammation mediates the association between traffic-related PM_2.5_ and AD
Huang et al. (2023, China) [45]	Longitudinal panel study	PM and NO_2_ (satellite-based random forests approach)	*BDNF* promoter methylation	MCI, AD	101	– Long-term exposures of PM and NO_2_ = significant changes in *BDNF* promoter methylation levels (non-linear curves with threshold effects)
Calderón-Garcidueñas et al. (2020, Mexico) [46]	Post-mortem study	PM	H3K9me2/me3 levels, *γ*-H2AX, hyperphosphorylated tau, and Aβ plaques	AD	23 (13 in Mexico and 10 in low-pollution areas)	– PM = impairs brain chromatin silencing, reduces DNA integrity = ↑ risk of developing AD in young individuals
Hong et al. (2025) [49]	Mendelian randomization, colocalization analysis, and enrichment analysis	515 compounds classified as EDCs, including bisphenol variants and phthalates	27 genes (*CCNE2, SEMA4G*, and *NDUFS2*)	AD	NA	– Involved in immune regulation and mitochondrial function (NK cell cytotoxicity and mitochondrial ribosomal pathways) – EDCs = immune and mitochondrial dysregulation => AD pathogenesis
An et al. (2021) [42]	Meta-analysis	BPS	Lipid, glucose, amino acid, nucleotide, and vitamin metabolism	Human diseases (AD, PD, and dementia)	NA	– BPS exposure => upregulated estrogen, downregulated androgen metabolism, and GSH deficiency
Cacciottolo et al. (2017, US) [38]	US-wide cohort from WHIMS and experimental mouse models	PM_2.5_ (exceeding EPA standards) and nPM	Aβ deposition, oligomers, and pro-amyloidogenic *APP* processing (sAPPβ/α ratio)	Cognitive impairment	3647	– Elevated Aβ and sAPPβ/*α* ratio = synaptic remodeling, effect on glutamate receptors, and neuronal atrophy – PM increased risk for global cognitive decline and ACD (adverse effect in *APOE-*ɛ4/4 carriers) – nPM exposure in mice = ↑ cerebral Aβ production

NO_2_: nitrogen dioxide, PM: particulate matter, NO_x_: nitrogen oxides, SO_2_: sulfur dioxide, EDCs: endocrine-disrupting chemicals, BPS: bisphenol S, nPM: nanosized particulate matter, miR: microRNA, PSD-95: postsynaptic density protein 95, NMDA: *N*-methyl-d-aspartate, NR2B: N-methyl-D-aspartate receptor subunit 2B, Aβ: amyloid-beta, NfL: neurofilament light chain, CpG: cytosine–phosphate–guanine, DNAm: DNA methylation, AD: Alzheimer’s disease, *BDNF*: brain-derived neurotrophic factor, NK: natural killer, GSH: glutathione, PD: Parkinson’s disease, *APP*: amyloid precursor protein, sAPPβ/*α* ratio: soluble amyloid precursor protein-beta to soluble amyloid precursor protein-alpha ratio, ACD: Alzheimer’s disease-related cognitive decline, *APP*-sw: Swedish mutant amyloid precursor protein.

## 4. Discussion

Overall, the available evidence suggests that long-term exposure to PM_2.5_ and NO_2_ is associated with increased dementia risk, with more limited evidence also suggesting a potential association with black carbon, whereas findings for PM_10_ and ozone remain inconsistent. Several factors may explain the heterogeneity across studies. First, exposure assessment differed substantially, including annual concentrations, multi-year averages, moving averages, baseline exposure, and life-course estimates. Second, studies used different exposure contrasts, such as fixed concentration increases, IQR increases, tertiles, and standard deviation increases, meaning that reported HRs cannot be directly compared based on their numerical magnitude alone. Third, geographical and temporal differences in pollution composition may influence toxicity. Finally, exposure estimates based on residential address may not capture individual mobility or exposures occurring outside the home, potentially introducing exposure misclassification.

These epidemiological associations are supported by biological plausibility, although the underlying pathways remain incompletely established. Air pollutants may reach or affect the brain through direct and indirect pathways. Smaller particles, specifically PM_2.5_ and ultrafine particles, may enter the central nervous system through the olfactory mucosa and nerves, where they could disrupt the blood–brain barrier, activate astrocytes, induce oxidative stress, and damage neurons. Additionally, pollutants entering the circulation from the lungs may also trigger systemic inflammation and impair the brain’s clearance mechanisms [50]. Together, these processes may contribute to neuronal injury and promote the accumulation of amyloid plaques and tau pathology, key hallmarks of dementia. Some evidence also suggests that vascular pathways may partly mediate air pollution’s effects on brain health.

Experimental and biomarker studies have also reported associations between PM_2.5_ and NO_2_ and amyloid-β deposition, tau hyperphosphorylation, and elevated neurofilament light chain concentrations, which are markers related to Alzheimer’s disease [3,38,46]. Metabolomic research has further identified that air pollution disrupts lipid and amino acid metabolism, with lipoprotein subclasses and glutathione-related metabolites indicating oxidative stress, mitochondrial dysfunction, and cerebrovascular dysfunction [39,40,41]. Epigenetic changes, including altered DNA methylation (*RBCK1* and *BDNF* promoter), histone modifications (H3K9 marks), and DNA damage (γ-H2AX), may contribute to neuroinflammation and genomic instability. These molecular alterations are likely to reinforce one another, creating a cycle of oxidative stress, chronic inflammation, and impaired protein clearance that accelerates amyloid and tau pathology [44,45,46]. Furthermore, the stronger effects observed among *APOE* ε4 carriers suggest that genetic susceptibility may amplify the neurotoxic effects of air pollution [3,38] (Table 5).

Evidence concerning environmental chemical pollutants was more limited and was dominated by cross-sectional studies. Among the exposure classes examined, plastic-associated endocrine-disrupting chemicals, heavy metals, and PFAS were associated with poorer cognitive function or dementia-related outcomes, whereas findings for pesticides and herbicides were inconsistent. For chemicals with established or suspected endocrine-disrupting activity, particularly bisphenols and phthalates, potential mechanisms include disruption of hormone signaling alongside oxidative stress, neuroinflammation, and metabolic dysfunction. Increased production of reactive oxygen species, including hydroxyl radicals (•OH), may overwhelm antioxidant defenses and promote chronic oxidative stress, which may subsequently activate neuroinflammatory pathways and contribute to neuronal injury and apoptosis [51].

Metabolomic research indicates that BPS disrupts glutathione, thiamine, and phosphatidylserine metabolism, potentially impairing neuronal activity. It may also affect estrogen and androgen metabolism, possibly through endocrine disruption, thus influencing brain homeostasis [42]. These findings are consistent with epidemiological research showing that higher BPS exposure was associated with Aβ+ status. Additionally, Mendelian randomization has identified several EDC-regulated genes related to AD that are crucial for neuronal survival (*CCNE2*), neuroimmune and synaptic signaling (*SEMA4G*), and mitochondrial oxidative phosphorylation (*NDUFS2*) [49]. In conclusion, chemicals with endocrine-disrupting activity, particularly bisphenols and phthalates, may contribute to neurodegeneration through interconnected metabolic, endocrine, and genetic pathways (Table 6). However, evidence for epigenetic mechanisms linking EDC exposure to dementia remains limited.

**Figure 2:**
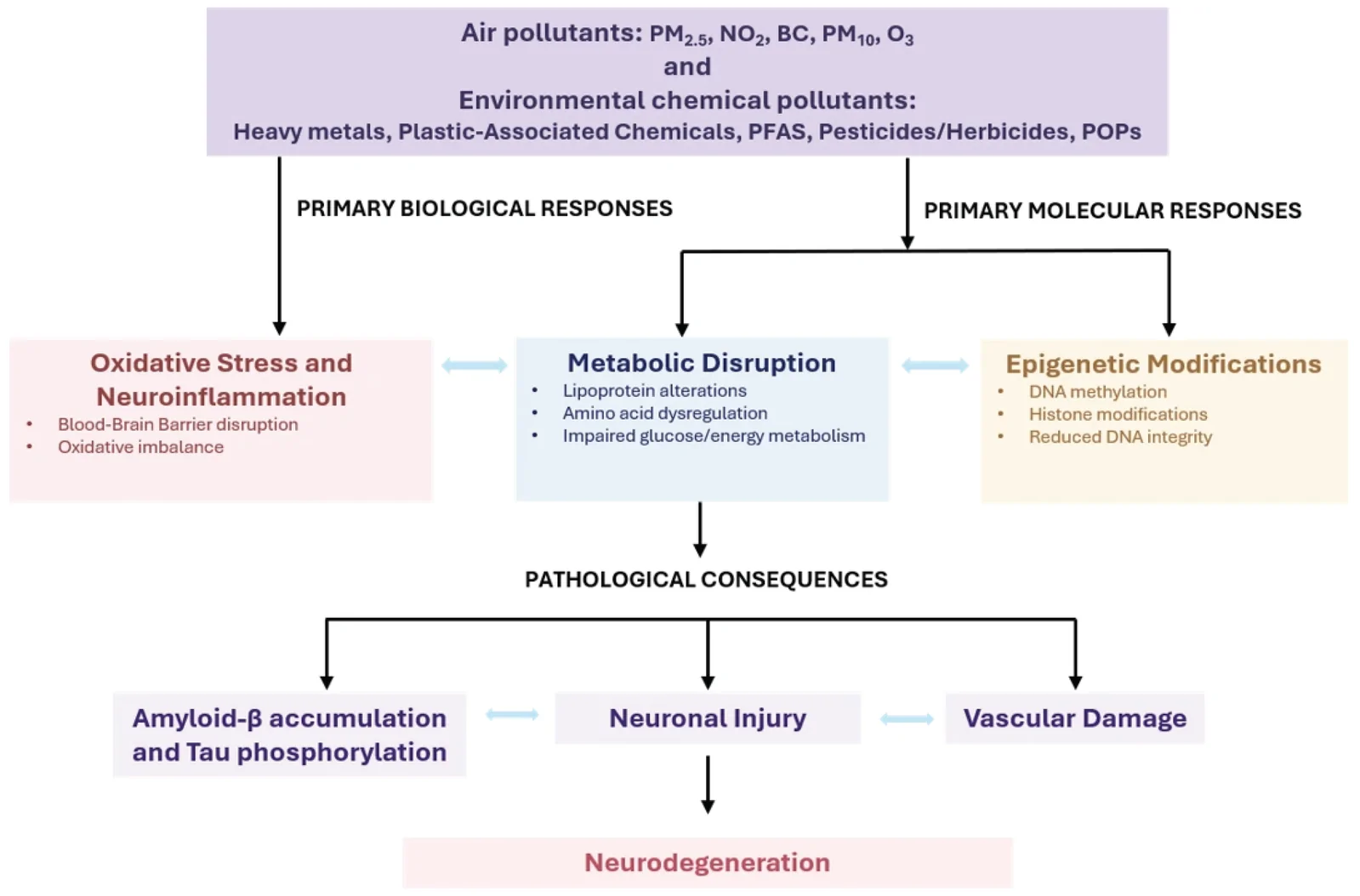
Proposed biological pathways linking air pollutants and environmental chemical pollutants to cognitive decline and dementia.

Although air pollutants and environmental chemical pollutants comprise chemically diverse exposure classes, current evidence suggests that they converge on a relatively small number of biological pathways, including oxidative stress, chronic neuroinflammation, mitochondrial dysfunction, vascular injury, and impaired lipid metabolism. Through these shared mechanisms, they may directly disrupt neuronal homeostasis and promote amyloid accumulation, tau pathology, and neurodegeneration, or indirectly contribute to cognitive impairment via vascular damage (Figure 2).

The current findings are consistent with the Lancet Commission Report (2024), which identified that long-term exposure to PM_2.5_ increases the risk of dementia. Our analysis also identifies evidence that higher exposure to air pollutants such as NO_2_ and black carbon is associated with increased dementia risk. In contrast, no clear associations were observed for NO_x_, PM_10_, or annual ozone. A recent systematic review in 2025 also supports these findings, indicating that long-term exposure to PM_2.5_, NO_2_, and black carbon was significantly associated with a higher risk of incident dementia [50].

This review has several limitations that should be considered when interpreting the overall findings and the certainty of the evidence. First, although multiple databases were searched, relevant studies may have been missed because of database coverage or language restrictions. Second, the search for mechanistic evidence was intended to provide contextual support for the epidemiological findings rather than to serve as a primary systematic review component. Therefore, we did not systematically search for or evaluate all biological mechanisms linking the exposures to the outcomes. This approach may have introduced confirmation bias. Third, some of the included studies may have used identical or overlapping cohorts. Where this occurred, we treated studies as independent evidence, which could exaggerate the apparent consistency or strength of the associations.

Our review extends the existing literature on environmental risk factors for dementia by incorporating emerging evidence on a broader range of environmental chemical pollutants. Current evidence indicates associations between exposure to several classes of environmental chemical pollutants and adverse cognitive outcomes. However, the absence of long-term cohort studies limits causal interpretation. Given the increased presence of these chemicals in food, packaging, and personal care products, this remains an under-researched but critical area for future investigation. Future research should move beyond individual pollutants towards integrated exposome approaches that capture cumulative lifetime environmental exposures. Large prospective cohorts incorporating repeated exposure assessment together with multi-omics technologies, including metabolomics, proteomics, and epigenomics, will help identify early biomarkers of neurodegeneration. The application of causal inference approaches, including Mendelian randomization and mediation analyses, may also strengthen causal understanding and support the development of targeted prevention strategies.

## Figures and Tables

**Table 1:** Characteristics of studies examining air pollution exposure and dementia risk.

Author (Year, Country)	Study Design	Population/Cases (Source)	Exposures	Outcomes
Wood et al. (2022, UK) [22]	Longitudinal cohort study	8525/389 (ELSA)	Baseline exposure (year 2004) to ambient NO_2_, PM_10_, PM_2.5_, and O_3_	Incident dementia
Ma et al. (2023, USA) [19]	Longitudinal cohort study	164,447/1394 (UK Biobank)	Annual mean levels of PM_2.5_, PM_coarse_, NO_2_, and PM_2.5_ absorbance	Incident dementia
Semmens et al. (2023, USA) [21]	Longitudinal cohort study	2564/324 (GEMS)	Long-term mean exposure to PM_2.5_ and NO_2_	Incident dementia, AD only, mixed VaD/AD
Zhang et al. (2023, USA) [25]	Longitudinal cohort study	27,857/4105 (EPOCH)	10-year mean total of PM_2.5_ and PM_2.5_ from 9 emission sources	Incident dementia
Jutila et al. (2024, UK) [18]	Longitudinal cohort study	572/67 (LBC1936)	Life-course PM_2.5_ and NO_2_	Incident dementia and AD
Oudin et al. (2024, Sweden) [20]	Longitudinal cohort study	70,057/409 (VIP)	Source-specific PM_2.5_ and PM_10_ (traffic exhaust, wood burning, and total PM)	Incident dementia
Wu et al. (2024, UK) [24]	Longitudinal cohort study	11,329/220 (CFAS II and Wales)	Annual concentrations of NO_2_, O_3_, PM_10_, and PM_2.5_	Incident dementia
Andersen et al. (2025, Denmark) [17]	Longitudinal cohort study	934,792/81,731 (Danish nationwide cohort)	Annual mean levels of PM_2.5_, NO_2_, and BC	Incident dementia
Wu et al. (2025, UK) [23]	Longitudinal cohort study	11,055/220 (CFAS II and Wales)	Air pollution (PM_10_, PM_2.5_, and NO_2_)	Incident dementia

ELSA: English Longitudinal Study of Ageing, NO_2_: nitrogen dioxide, PM: particulate matter, O_3_: ozone, GEMS: Ginkgo Evaluation of Memory Study, AD: Alzheimer’s disease, VaD: vascular dementia, EPOCH: Environmental Predictors of Cognitive Health and Aging, VIP: Västerbotten Intervention Program, LBC1936: Lothian Birth Cohort 1936, CFAS: Cognitive Function and Ageing Study, BC: black carbon.

**Table 2:** Main findings of studies examining air pollution exposure and dementia risk.

Author (Year, Country)	Window	Duration	Exposures	Mean Exposure	Outcomes	HR	Unit	Key Findings
Wood et al. (2022, UK) [22]	Estimate of baseline exposure (2004)	8.45 years	PM_2.5_	12.13 (SD, 4.41) µg/m^3^	Incident dementia	1.10 (0.88–1.37)	per 10 µg/m^3^	PM_2.5_ and ozone showed a non-significant risk increase; NO_2_ and PM_10_ showed no association.
			NO_2_	25.5 (SD, 13.7) µg/m^3^		0.97 (0.89–1.05)	per 10 µg/m^3^	
			PM_10_	19.15 (SD, 10.51) µg/m^3^		0.98 (0.89–1.08)	per 10 µg/m^3^	
			O_3_	46.88 (SD, 13.53) µg/m^3^		1.01 (0.94–1.09)	per 10 µg/m^3^	
Ma et al. (2023, USA) [19]	Average values of 2005–2007 and 2010	8.9 years	PM_2.5_	9.9 (SD, 1.0) µg/m^3^	Incident all-cause dementia	1.10 (1.04–1.17)	per 1 µg/m^3^	Higher exposure to PM_2.5_ and NO_2_ was associated with higher dementia risk. PM_2.5_ absorbance and PM_coarse_ showed no association.
	Average values of 2010		NO_2_	28.1 (SD, 8.7) µg/m^3^		1.09 (1.05–1.14)	per 5 µg/m^3^	
	Average values of 2010		PM_coarse_	6.4 (SD, 0.9) m		0.99 (0.94–1.06)	per 0.1 m	
	Average values of 2010		PM_2.5_ absorbance	1.16 (SD, 0.26) µg/m^3^		1.03 (1.01–1.06)	per 1 µg/m^3^	
Semmens et al. (2023, USA) [21]	mean PM_2.5_ exposure in the 5, 10, 15, and 20 years prior to enrolment	5.7 years	PM_2.5_	12 to 18 µg/m^3^	Incident dementia, AD, and VaD and AD mixed	1.2 (1.05–1.37) 20 years prior to enrolment	per 2 µg/m^3^	Long-term exposure to PM_2.5_ and NO_2_ was associated with higher dementia risk and increased risk of mixed VaD/AD but not AD alone.
	mean NO_2_ exposure in the 5 and 10 years prior to enrolment		NO_2_	12 to 25 ppb		1.18 (1.02–1.37) 10y	per 5 ppb	
						1.18 (1.01–1.37) 5y		
Zhang et al. (2023, USA) [25]	10-year mean	10.2 years	Total PM_2.5_	median (IQR) 11.2 (9.5–13.2)	Incident dementia	1.08 (1.01–1.17)	per IQR increase	Higher concentrations of total PM_2.5_ increased dementia risk; agricultural and wildfire PM_2.5_ showed the strongest associations with dementia risk.
			PM_2.5_ from Agriculture	1.1 (0.6–1.7)		1.17 (1.05–1.30)	per IQR increase	
			PM_2.5_ from Wildfires	1.0 (0.7–1.3)		1.04 (1.01–1.07)	per IQR increase	
Jutila et al. (2024, UK) [18]	Life-course exposure: 1935, 1950, 1970, 1980, 1990, 2001, and 2007	11.26 years	PM_2.5_	mean estimated exposure to PM2.5 decreased 31.45 (SD, 14.03)−5.96 (SD, 0.25)	Incident dementia and AD	1.18 (0.92–1.51)	per 1 SD	In utero and early-life PM_2.5_ and NO_2_ exposure were associated with higher dementia and AD risk; cumulative exposure to PM_2.5_ was associated with higher dementia and AD risk.
			NO_2_	mean exposure increased 14.39 (SD, 5.35)−20.86 (SD, 5.39)		1.15 (0.89–1.49)	per 1 SD	
Oudin et al. (2024, Sweden) [20]	Moving-average exposure (Lag 1–5 years/Lag 6–10 years before event)	12.6 years	total PM_10_	9.5 (SD, 1.38) lag1–5	Incident dementia	0.83 (0.61–1.11) lag1–5	tertiles	No positive association between total PM_10_ or total PM_2.5_ and incident dementia. Traffic-related PM_10_ was associated with higher dementia risk.
				9.76 (SD, 1.39) lag6–10		0.82 (0.61–1.11) lag6–10		
			total PM_2.5_	5.61 (SD, 0.81) lag1–5		0.9 (0.68–1.38) lag1–5	tertiles	
				5.75 (SD, 0.81) lag6–10		0.78 (0.57–1.05) lag6–10		
			PM_10_—Traffic	0.51 (SD, 0.61) lag1–5		1.35 (1.00–1.82) lag1–5	tertiles	
				0.49 (SD, 0.6) lag6–10		1.02 (0.75–1.37) lag6–10		
			PM_2.5_—Exhaust	0.1 (SD, 0.12) lag1–5		1.33 (0.98–1.79) lag1–5	tertiles	
				0.11 (SD, 0.13) lag6–10		0.99 (0.74–1.38) lag6–10		
			PM_2.5_—Wood burning	0.78 (SD, 0.33) lag1–5		0.67 (0.51–0.89) lag1–5	tertiles	
				0.76 (SD, 0.33) lag6–10		0.76 (0.54–1.06) lag6–10		
Wu et al. (2024, UK) [24]	Annual concentration (2012)	2 years	PM_2.5_	11.9 (IQR, 3.5) µg/m^3^	Incident dementia	1.06 (0.7–1.62) CFASII	per 1 µg/m^3^	Associations were inconsistent and largely null.
						0.82 (0.55–1.23) CFAS Wales		
			PM_10_	15.5 (IQR, 4.2) µg/m^3^		1.06 (0.8–1.4) CFASII	per 1 µg/m^3^	
						0.95 (0.82–1.12) CFAS Wales		
			NO_2_	21.1 (IQR, 14.2) µg/m^3^		1.06 (1.01–1.1) CFASII	per 1 µg/m^3^	
						0.96 (0.88–1.04) CFAS Wales		
			O_3_	39.4 (IQR, 6.4) µg/m^3^		0.86 (0.77–0.97) CFASII	per 1 µg/m^3^	
						1.16 (0.99–1.37) CFAS Wales		
Andersen et al. (2025, Denmark) [17]	Annual mean level (2010) at baseline (2000) address	11.6 years	PM_2.5_	12.5 (SD, 1.51) µg/m^3^	Incident dementia	1.14 (1.12–1.16)	per 1.9 µg/m^3^	PM_2.5_, NO_2_, and BC increased dementia risk, particularly among older and socioeconomically disadvantaged populations.
			NO_2_	20.6 (SD, 7.85) µg/m^3^		1.25 (1.22–1.28)	per 10.2 µg/m^3^	
			BC	1.3 (SD, 0.36) ×10^−5^/m		1.23 (1.2–1.26)	per 0.5 × 10^−5^/m	
Wu et al. (2025, UK) [23]	Annual mean concentrations (2012)	2 years	Air pollution (mixed PM_10_, PM_2.5_, and NO_2_)	NO_2_ 19.2 (SD, 8.7)	Incident dementia	0.91 (0.78–1.07)	per 1 unit	Air pollution mixtures showed no clear effect.
				PM_10_ 16.1 (SD, 2)				
				PM_2.5_ 10.3 (SD, 2.7)				

NO_2_: nitrogen dioxide, PM: particulate matter, O_3_: ozone, AD: Alzheimer’s disease, VaD: vascular dementia, BC: black carbon, SD: standard deviation, ppb: parts per billion, IQR: interquartile range.

**Table 3:** Characteristics and main findings of studies examining environmental chemical pollutants and dementia-related outcomes.

Author (Year, Country)	Study Design	Population/Cases (Source)	Exposures	Outcomes	Key Findings
Li et al. (2023, China) [34]	Cross-sectional	N = 514 (Beijing cohort)	14 metals in blood (Al, Cd, Co, Cr, Cu, Li, Mn, Mo, Ni, Pb, Se, Sr, V, and Zn)	Cognitive function: MMSE test	Blood Cu [β (95%CI): −3.73 (−6.42, −1.03)] and Pb [β (95%CI): −0.79 (−1.26, −0.32)] levels were negatively associated with cognitive function, while Se was beneficial [β (95%CI): 2.97 (1.23, 4.70)]. Se was found to attenuate the neurotoxicity of a Cu–Pb mixture.
Fu et al. (2024, USA) [33]	Cross-sectional	N = 811 (NHANES)	Blood levels of Pb, Cd, Hg, Se, Cu, and Zn	Cognitive function: IRT, DRT, AFT, and DSST tests	High blood levels of Pb, Cd, Hg, and Cu were negatively correlated with cognitive function, while Se and Zn were protective. Joint exposure showed mixed linear associations across different cognitive tests.
Liu et al. (2024, China) [32]	Cross-sectional	N = 2445/489 (SAD)	21 metals (As, Se, Zn, Fe, Li, Al, Ti, V, Cr, Mn, Co, Ni, Cu, Rb, Sr, Sn, Ba, Mo, Cd, Ti, and Pb) measured in urine; diet behaviors collected by SFQ	MCI diagnosis: Mini-Cog test	Urinary As [OR (95%CI): 1.19 (1.5, 1.42)] and Cd [OR (95%CI): 1.32 (1.01, 1.74)] concentrations were positively associated with increased MCI risk.
Zhou et al. (2025, USA) [26]	Cross-sectional	N = 959 (NHANES)	Pb, Cd, Hg, Mn, and Se in blood	Neurological injury biomarker: sNfL; cognitive function: AFT, CERAD, and DSST tests	Metal mixtures were associated with neuroaxonal injury biomarkers [OR (95%CI): 0.051 (0.025, 0.090)], with Pb [β (95%CI): 0.14 (0.08, 0.20)] and Cd [β (95%CI): 0.14 (0.07, 0.20)] being harmful and Se [β (95%CI): −0.36 (−0.67, −0.05)] showing protective associations.
Zou et al. (2024, USA) [30]	Cross-sectional	N = 1364 (NHANES)	Herbicide (urinary 2,4-D concentrations)	Cognitive function: CERAD, DSST, and AFT tests	There is a U-shaped relationship between human urinary 2,4-D concentrations and cognitive impairment, especially in males.
Ruiz-Gonzalez et al. (2024, Spain) [31]	Cross-sectional	N = 591,736/40,044 (Spain cohort)	Residential region categorized by environmental pesticide use (high/low)	AD diagnosis (ICD9 and ICD10)	Residence in high-pesticide-use regions was associated with a higher prevalence of AD [OR (95%CI): 1.64 (1.41, 1.89)].
Canovas et al. (2025, Australia) [29]	Cross-sectional	N = 191 (AIBL)	12 phthalate metabolites and 5 bisphenols measured in the urine	AD biomarker: amyloid-β (Aβ); cognitive status (MCI/AD)	Low MEHP and high BPS were associated with Aβ+. A mixture of plastic-associated chemicals was significantly associated with clinical MCI/AD status [OR (95%CI): 1.30 (1.03, 1.64)], but not with Aβ+.
Du et al. (2025, USA) [28]	Cross-sectional	N = 856 (NHANES)	Mixed phenols (BPA and TCS), parabens (MPB and EPB), and phthalate (MECP, MEOH, MEHP, MIBP, and MBZP) metabolites measured in the urine	Cognitive function: IRT, AFT, and DSST tests	Mixed exposure was positively associated with cognitive impairment in males for IRT [OR (95%CI): 1.77 (1.04, 3.02)] and global tests [OR (95%CI): 2.32 (1.15, 4.67)]. Phthalate metabolites (MEHP and MECP) played a dominant role in these negative effects.
Lefevre-Arbogast et al. (2025, France) [27]	Longitudinal cohort studies (duration: 17 years)	N = 515/105 (3C)	Plasma concentrations of 15 PCBs, 12 OCPs, and 1 BFR, as well as a POP composite score	Incident dementia, cognitive decline, and brain atrophy (MTL volume)	Neither individual POPs nor the total POP score were significantly and consistently associated with dementia-related outcomes. Highly chlorinated PCBs were associated with dementia risk [HR (95% CI): 1.51 (1.03, 2.21)] and medial temporal lobe atrophy [β (95%CI): −0.28 (−0.49, −0.06)] in *APOE*-ε4 carriers.
Lefevre-Arbogast et al. (2024, France) [35]	Longitudinal cohort studies (duration: 17 years)	N = 1288/314 (3C)	Dietary exposure to 167 chemical contaminants (4 food additives, 47 pesticides, 18 mycotoxins, 16 metal trace elements, 17 dioxins/furans, 18 PCBs, 14 brominated flame retardants, 21 PAHs, and 12 PFAS) measured by FFQ and 24 HDR	Incident dementia	Higher dietary exposure to 85 contaminants (out of 167 studied) was linked to increased dementia risk, especially among high-fat diet consumers. The strongest associations were for α-HBCDD and PFOS (HR = 1.50 and 1.43, respectively). A signature of four PFAS, two BFRs, two mycotoxins, and nitrites was associated with higher dementia risk in individuals on a high-fat diet [HR (95% CI): 3.5 (1.9, 6.5)].
Pan et al. (2026, USA) [36]	Cross-sectional	N = 65 (USA firefighters)	24 PFAS measured in blood	Global cognition and processing speed: NIH Toolbox Cognition Battery	PFOS was significantly negatively associated with processing speed scores [β (95%CI): −0.58 (−0.91, −0.24)]. No significant associations were found between individual PFAS, the 6-PFAS mixture, or the 24-PFAS mixture and global cognition.

NHANES: National Health and Nutrition Examination Survey, SAD: Shenzhen Age-related Disorder, AIBL: Australian Imaging, Biomarker & Lifestyle, 3C: The Three-City study, β: linear regression coefficients, CI: confidence interval, OR: odds ratio, HR: hazard ratios, Al: aluminum, Cd: cadmium, Co: cobalt, Cr: chromium, Cu: copper, Li: lithium, Mn: manganese, Mo: molybdenum, Ni: nickel, Pb: lead, Se: selenium, Sr: strontium, V: vanadium, Zn: zinc, Hg: mercury, As: arsenic, Fe: iron, Ti: titanium, Rb: rubidium, Sn: tin, Ba: barium, 2,4-D: 2,4-dichlorophenoxyacetic acid, BPA: bisphenol A, TCS: triclosan, MPB: methyl paraben, EPB: ethyl paraben, MEHP: mono(2-ethylhexyl) phthalate, MIBP: mono-isobutyl phthalate, MBZP: mono-benzyl phthalate, PCB: polychlorinated biphenyl, OCP: organochlorine pesticide, BFR: brominated flame retardant, POP: persistent organic pollutant, PAH: polycyclic aromatic hydrocarbon, PFAS: per- and polyfluoroalkyl substances, MMSE: the Mini-Mental State Examination, IRT: Item Response Theory, DRT: Digit Recall Test, AFT: Animal Fluency Test, DSST: Digit Symbol Substitution Test, Aβ: amyloid-beta, sNfL: serum neurofilament light chain, MCI: mild cognitive impairment, AD: Alzheimer’s disease, CERAD: Consortium to Establish a Registry for Alzheimer’s Disease, MTL: medial temporal lobe, NIH: National Institutes of Health, SFQ: Short Food Questionnaire, FFQ: Food Frequency Questionnaire, HDR: 24-hour dietary recall.

**Table 5:** Summary of biomarkers associated with air pollution and dementia.

Category	Biomarker	Evidence of Pollutant Disruption/Major Changes	Proposed Link to Dementia
**Protein/Neuropathology Biomarkers**	Amyloid-β	• Greater exposure to NO_2_ and PM_2.5_ absorbance increase brain Aβ deposition levels [3]. • Chronic exposure to nPM increases Aβ deposits, the sAPPβ/*α* ratio, and neurotoxic oligomers in brain tissue [38].	Aβ accumulation outside and between nerve cells (neurons) is a hallmark of AD. Increased amyloidogenic *APP* processing is associated with impaired synaptic plasticity, glutamatergic dysfunction, and neuronal degeneration.
	Hyperphosphorylated Tau	• Exposure to PM_2.5_ is associated with increased tau phosphorylation [46].	Tau tangles accumulating inside neurons are a hallmark of AD.
	Neurofilament light chain (NfL)	• Greater exposure to PM_2.5_ and PM_10_ increases CSF NfL levels [3].	NfL is a highly sensitive biomarker for neurological diseases; elevated levels indicate neurological injury.
**Metabolomic Biomarkers**	Lipids and lipoprotein subclasses	• Pollution-related metabolic signature scores (NO_2_, PM_2.5_, PM_10_, NO_x_) were associated with a higher risk of dementia. Metabolites from lipoprotein subclasses/M-VLDL-FC played essential mediating roles [39]. • Increased level of triglycerides reduces dementia risk, slows the decline in composite cognition and memory over time [41].	Lipoprotein alterations may disrupt brain lipid signaling via the VLDL receptor, impairing neuronal membrane integrity, synaptic plasticity, and cognitive aging.
	Cysteinylglycine disulfide, a diglyceride, and a dicarboxylic acid/amino acid	• Long-term exposure to PM_2.5_ is negatively associated with circulating levels of this metabolite [40].	Derived from the degradation of glutathione, they reflect disruptions in oxidative stress, lipid metabolism, and mitochondrial energy production.
**Epigenetic Biomarkers**	MicroRNAs (miR-337-5p)	• Co-exposure to PM_2.5_ and SO_2_ alters miR-337-5p [43].	Altered miR-337-5p may influence tau-related pathways and contribute to tau hyperphosphorylation, neuronal apoptosis, and synaptic dysfunction.
	Histone H3K9me2/H3K9me3	• Exposure to PM_2.5_ reduces these repressive marks in the prefrontal cortex [46].	Loss of these marks causes global chromatin relaxation, genome instability, and aberrant gene transcription.
	γ-H2AX (DNA damage)	• Exposure to PM_2.5_ significantly increases nuclear γ-H2AX intensity [46].	An early cellular response to DNA double-strand breaks indicates reduced DNA integrity, which can lead to neurotoxicity.
	*BDNF* promoter methylation	• Long-term exposure to PM_1_, PM_2.5_, PM_10_, and NO_2_ causes hypermethylation of the peripheral *BDNF* promoter [45].	Methylation of the *BDNF* gene was associated with AD, which reduces the mRNA or protein levels in the brain, especially in the hippocampus or frontal cortex.
	DNA methylation	• Traffic-related PM_2.5_ altered CpG methylation (cg25433380 in the intergenic region and cg10495669 in the *RBCK1* gene) but were not found to be associated with any neuropathology markers [44].	*RBCK1* is a neuroinflammation gene; its overactivation promotes inflammatory pathways linked to AD.
**Genetic Biomarkers**	*APOE* ε4 allele	• Exposure to NO_2_ and PM_2.5 absorbance_ is associated with a significantly greater increase in brain Aβ deposition levels [3]. • PM exposure has a significantly stronger adverse effect on cognitive decline in *APOE *ε4 carriers [38].	The *APOE *ε4 gene variant is the strongest known genetic risk factor for AD.

NO_2_: nitrogen dioxide, PM: particulate matter, Aβ: amyloid-beta, nPM: nanosized particulate matter, sAPPβ/*α* ratio: soluble amyloid precursor protein-beta to soluble amyloid precursor protein-alpha ratio, APP: amyloid precursor protein, CSF: cerebrospinal fluid, NfL: neurofilament light chain, VLDL: very-low-density lipoprotein, M-VLDL-FC: medium very-low-density lipoprotein free cholesterol, SO_2_: sulfur dioxide, miR: microRNA, CpG: cytosine–phosphate–guanine.

**Table 6:** Summary of molecular biomarkers and pathways associated with environmental chemical pollutants exposure and dementia.

Category	Biomarker	Evidence of Pollutant Disruption/Major Changes	Proposed Link to Dementia
**Metabolomic Biomarkers**	Glutathione (GSH)	High concentrations of Bisphenol S (BPS) downregulate glutathione synthetase, leading to a significant reduction in GSH levels [42].	GSH deficiency is directly related to the development and aggravation of AD, Parkinsonism, and dementia.
	Thiamine (Vitamin B1)	BPS interferes with thiamine metabolism, leading to reduced thiamine pyrophosphate levels [42].	Thiamine is critical for brain glucose utilization; deficiency leads to reduced glucose use in the brain, common in AD patients.
	Phosphatidylserine	BPS interferes with the conversion of amino acids into phospholipids such as phosphatidylserine [42].	Phosphatidylserine is essential for maintaining cell membrane fluidity and is used to treat both AD and non-AD dementia.
	Estrone/Testosterone	BPS exposure upregulates estrogen synthesis while downregulating androgen metabolism in humans and mice [42].	Hormonal imbalances and cholesterol dysfunction cause endocrine disorders that can disrupt neurohomeostasis.
**Genetic Biomarkers**	*CCNE2, SEMA4G*, and *NDUFS2*	27 EDC-regulated genes showed significant causal associations with AD via MR analysis. Enrichment analysis showed their involvement in immune and mitochondrial pathways. *CCNE2, SEMA4G*, and *NDUFS2* are the strongest candidates among the genes [49].	These genes implicate biological pathways involved in neuronal survival (*CCNE2*), neuroimmune and synaptic signaling (*SEMA4G*), and mitochondrial oxidative phosphorylation (*NDUFS2*), supporting neuroinflammation and mitochondrial dysfunction as potential pathways linking EDCs to dementia.

BPS: bisphenol S, GSH: glutathione, AD: Alzheimer’s disease, EDCs: endocrine-disrupting chemicals, MR: Mendelian randomization.

## Data Availability

No new data were created or analyzed in this study. Data sharing is not applicable to this article.

## Supplementary Materials

The following supporting information can be downloaded at: https://www.mdpi.com/article/10.3390/ijms27188224/s1.
