# Supplementary material for: Environmental Pollution, Biomarkers, and Dementia: An Evidence Review
Source: Int J Mol Sci. 2026 Sep 15;27(18):8224. doi: 10.3390/ijms27188224 (PMC13607459; doi:10.3390/ijms27188224)
Supplement: Supplementary file 1 [file ijms-27-08224-s001.zip › ijms-4471126-supplementary.pdf]

Supplementary Methods

PubMed Full Search Terms:

(("phthalates"[Title/Abstract] OR "heavy metals"[Title/Abstract] OR "air pollution"[Title/Abstract] OR "PM2.5"[Title/Abstract] OR "PM10"[Title/Abstract] OR "NO2"[Title/Abstract] OR "pesticides"[Title/Abstract] OR "Endocrine-disrupting chemicals"[Title/Abstract] OR "PFAS"[Title/Abstract] OR "per and polyfluoroalkyl substances"[Title/Abstract]) AND ("Alzheimer Disease"[Title/Abstract] OR "cognitive impairment"[Title/Abstract] OR "MCI"[Title/Abstract] OR "dementia"[Title/Abstract] OR "mild cognitive impairment"[Title/Abstract])) AND ((fft[Filter]) AND (humans[Filter]) AND (2020/1/1:2026/3/30[pdat]) AND (english[Filter]))

Embase Full Search Terms:

1 (phthalates or heavy metals or air pollution or PM2 5 or PM10 or NO2 or pesticides or endocrine disrupting chemicals or PFAS or "per- and polyfluoroalkyl substances").ti,ab.

2 (Alzheimer disease or cognitive impairment or MCI or dementia or mild cognitive impairment).ti,ab.

3 1 and 2

4 limit 3 to (full text and human and english language and "remove preprint records" and yr="2020 - 2026")

Supplementary Table S1. Quality Assessment for Cohort Studies.

| Study (Year) | Selection | | | | Comparability | Outcome | | | Total |
|---|---|---|---|---|---|---|---|---|---|
| | **1** | **2** | **3** | **4** | **5** | **6** | **7** | **8** | |
| Wood et al. (2022) [1] | 1 | 1 | 1 | 1 | 2 | 0 | 1 | 0 | 7/9 |
| Ma et al. (2023) [2] | 1 | 1 | 1 | 1 | 2 | 1 | 1 | 1 | 9/9 |
| Semmens et al. (2023) [3] | 1 | 1 | 1 | 1 | 2 | 1 | 1 | 1 | 9/9 |
| Zhang et al. (2023) [4] | 1 | 1 | 1 | 1 | 2 | 1 | 1 | 1 | 9/9 |
| Jutila et al. (2024) [5] | 1 | 1 | 1 | 1 | 2 | 1 | 1 | 0 | 8/9 |
| Oudin et al. (2024) [6] | 1 | 1 | 1 | 1 | 2 | 1 | 1 | 1 | 9/9 |
| Wu et al. (2024) [7] | 1 | 1 | 1 | 1 | 2 | 1 | 0 | 0 | 7/9 |
| Andersen et al. (2025) [8] | 1 | 1 | 1 | 1 | 2 | 1 | 1 | 1 | 9/9 |
| Wu et al. (2025) [9] | 1 | 1 | 1 | 1 | 2 | 1 | 0 | 0 | 7/9 |
| Lefevre-Arbogast et al. (2025) [10] | 1 | 1 | 1 | 1 | 2 | 1 | 1 | 1 | 9/9 |
| Lefevre-Arbogast et al. (2024) [11] | 1 | 1 | 1 | 1 | 1 | 1 | 1 | 1 | 8/9 |

1. Representativeness of the exposed cohort; 2. Selection of the non-exposed cohort; 3. Ascertainment of exposure; 4. Demonstration that outcome of interest was not present at start of study; 5. Comparability of cohorts on the basis of the design or analysis; (2 stars max); 6. Assessment of outcome; 7. Was follow-up long enough for outcomes to occur; 8. Adequacy of follow-up of cohorts.

Supplementary Table S2. Quality Assessment for Cross-sectional Studies.

| **Study (Year)** | **Study Sample Selection** | | **Assessment of Exposure and Outcome** | | **Outcome** | | **Total** |
|---|---|---|---|---|---|---|---|
| | **1** | **2** | **3** | **4** | **5** | **6** | |
| Li et al. (2023) [12] | 1 | 1 | 2 | 2 | 2 | 1 | 9/9 |
| Fu et al. (2024) [13] | 1 | 1 | 2 | 2 | 2 | 1 | 9/9 |
| Liu et al. (2024) [14] | 1 | 1 | 2 | 2 | 2 | 1 | 9/9 |
| Zhou et al. (2025) [15] | 1 | 1 | 2 | 2 | 2 | 1 | 9/9 |
| Zou et al. (2024) [16] | 1 | 1 | 2 | 2 | 2 | 1 | 9/9 |
| Ruiz-Gonzalez et al. (2024) [17] | 1 | 1 | 1 | 2 | 2 | 1 | 8/9 |
| Canovas et al. (2025) [18] | 0 | 1 | 2 | 2 | 2 | 1 | 8/9 |
| Du et al. (2025) [19] | 1 | 1 | 2 | 2 | 2 | 1 | 9/9 |
| Pan et al. (2026) [20] | 0 | 0 | 2 | 2 | 2 | 1 | 7/9 |

1. Representativeness of the study sample; 2. Sample size; 3. Assessment of the exposure(s); 4. Assessment of the outcome(s); 5. Adjustment for confounder(s); 6. Assessment for confounder(s)

**NEWCASTLE - OTTAWA QUALITY ASSESSMENT SCALE [21]**

**COHORT STUDIES**

Note: A study can be awarded a maximum of one star for each numbered item within the Selection and Outcome categories. A maximum of two stars can be given for Comparability

**Selection**

1) Representativeness of the exposed cohort

a) truly representative of the average _______________ (describe) in the community *

b) somewhat representative of the average ______________ in the community *

c) selected group of users e.g. nurses, volunteers

d) no description of the derivation of the cohort

2) Selection of the non-exposed cohort

a) drawn from the same community as the exposed cohort *

b) drawn from a different source

c) no description of the derivation of the non-exposed cohort

3) Ascertainment of exposure

a) secure record (e.g. surgical records) *

b) structured interview *

c) written self-report

d) no description

4) Demonstration that outcome of interest was not present at start of study

a) yes *

b) no

**Comparability**

1) Comparability of cohorts on the basis of the design or analysis

a) study controls for _____________ (select the most important factor) *

b) study controls for any additional factor ◎ (This criteria could be modified to indicate specific control for a second important factor.)

**Outcome**

1) Assessment of outcome

a) independent blind assessment *

b) record linkage *

c) self-report

d) no description

2) Was follow-up long enough for outcomes to occur

a) yes (select an adequate follow-up period for outcome of interest) *

b) no

3) Adequacy of follow-up of cohorts

a) complete follow-up - all subjects accounted for *

b) subjects lost to follow up unlikely to introduce bias - small number lost - > ____ % (select an adequate %) follow up, or description provided of those lost) *

c) follow up rate < ____% (select an adequate %) and no description of those lost

d) no statement

## NEWCASTLE - OTTAWA QUALITY ASSESSMENT SCALE [22]

## (adapted for cross-sectional studies)

| Domain | Item | Answer (only 1 per item possible) | Rating | Awarded Star(s) |
|---|---|---|---|---|
| **STUDY SAMPLE SELECTION (max. 2 stars)** | **1. Representativeness of the study sample** | a) Truly representative of the target population (all subjects or random sampling). | ✸ | ___ /1 star |
| | | b) Somewhat representative of the target population (non-random sampling). | ✸ | |
| | | c) No representative of the target population (selected group of users). | - | |
| | | d) No description of the sampling strategy. | - | |
| | **2. Sample size** | a) Justified and satisfactory. | ✸ | ___ /1 star |
| | | b) Not justified or unsatisfactory. | - | |
| **ASSESSMENT of EXPOSURE and OUTCOME (max. 4 stars)** | **3. Assessment of the exposure(s)** | a) Gold-standard assessment tool. | ✸✸ | ___ / 2 stars |
| | | b) Acceptable assessment tool. | ✸ | |
| | | c) Non-acceptable assessment methods. | - | |
| | | d) No description of the assessment tool. | - | |
| | **4. Assessment of the outcome(s)** | a) Gold-standard assessment tool. | ✸✸ | ___ / 2 stars |
| | | b) Acceptable assessment tool. | ✸ | |
| | | c) Non-acceptable assessment methods. | - | |
| | | d) No description of the measurement tool. | - | |
| **CONFOUNDING FACTORS (max. 3 stars)** | **5. Adjustment for confounder(s)** | a) The study estimates are adjusted for all the most important confounding factors, and not adjusted for potential mediator(s). | ✸✸ | ___ / 2 stars |
| | | b) The study estimates are adjusted for all the most important confounding factors, but also for potential mediator(s). | ✸ | |
| | | c) The study estimates are adjusted for some but not all the most important confounding factors. | ✸ | |
| | | d) The study estimates are not adjusted for any of the most important confounding factors or no description is provided. | - | |
| | **6. Assessment of confounder(s)** | a) Most of the confounding factors controlled for were measured through gold standard assessment methods. | ✸ | ___ / 1 star |
| | | b) Most of the confounding factors controlled for were measured through acceptable assessment methods. | ✸ | |
| | | c) Most of the confounding factors controlled for were not measured through at least acceptable assessment methods. | - | |
| | | d) No confounding factors controlled for. | - | |
| **Total score** | | | | **___ / 9 stars** |

Reference


1. Wood, D.; Evangelopoulos, D.; Beevers, S.; Kitwiroon, N.; Katsouyanni, K., Exposure to Ambient Air Pollution and the Incidence of Dementia in the Elderly of England: The ELSA Cohort. *Int J Environ Res Public Health* **2022,** 19, (23).
2. Ma, H.; Li, X.; Zhou, T.; Wang, M.; Heianza, Y.; Qi, L., Long-term exposure to low-level air pollution, genetic susceptibility and risk of dementia. *International Journal of Epidemiology* **2023,** 52(3), 738-748.
3. Semmens, E. O.; Leary, C. S.; Fitzpatrick, A. L.; Ilango, S. D.; Park, C.; Adam, C. E.; DeKosky, S. T.; Lopez, O.; Hajat, A.; Kaufman, J. D., Air pollution and dementia in older adults in the Ginkgo Evaluation of Memory Study. *Alzheimer's and Dementia* **2023,** 19(2), 549-559.
4. Zhang, B.; Weuve, J.; Langa, K. M.; D'Souza, J.; Szpiro, A.; Faul, J.; Mendes de Leon, C.; Gao, J.; Kaufman, J. D.; Sheppard, L.; Lee, J.; Kobayashi, L. C.; Hirth, R.; Adar, S. D., Comparison of Particulate Air Pollution From Different Emission Sources and Incident Dementia in the US. *JAMA Intern Med* **2023,** 183, (10), 1080-1089.
5. Jutila, O. E. I.; Mullin, D.; Vieno, M.; Tomlinson, S.; Taylor, A.; Corley, J.; Deary, I. J.; Cox, S. R.; Baranyi, G.; Pearce, J.; Luciano, M.; Karlsson, I. K.; Russ, T. C., Life-course exposure to air pollution and the risk of dementia in the Lothian Birth Cohort 1936. *Environmental Epidemiology* **2024,** 9(1), e355.
6. Oudin, A.; Raza, W.; Flanagan, E.; Segersson, D.; Jalava, P.; Kanninen, K. M.; Rönkkö, T.; Giugno, R.; Sandström, T.; Muala, A.; Topinka, J.; Sommar, J., Exposure to source-specific air pollution in residential areas and its association with dementia incidence: a cohort study in Northern Sweden. *Sci Rep* **2024,** 14, (1), 15521.
7. Wu, Y. T.; Kitwiroon, N.; Beevers, S.; Barratt, B.; Brayne, C.; Cerin, E.; Franklin, R.; Houlden, V.; Woods, B.; Zied Abozied, E.; Prina, M.; Matthews, F., The longitudinal associations between ambient air pollution exposure and dementia in the UK: results from the cognitive function and ageing study II and Wales. *BMC Public Health* **2024,** 24, (1), 1233.
8. Andersen, Z. J.; Lim, Y. H.; Zhang, J.; Tuffier, S.; Cole-Hunter, T.; Bergmann, M.; Loft, S.; Mortensen, L. H.; Chen, J.; Stafoggia, M.; de Hoogh, K.; Katsouyanni, K.; Vienneau, D.; Rodopoulou, S.; Samoli, E.; Bauwelinck, M.; Klompmaker, J. O.; Atkinson, R.; Janssen, N. A. H.; Oftedal, B.; Renzi, M.; Forastiere, F.; Strak, M.; Thygesen, L. C.; Zaninotto, P.; Brunekreef, B.; Hoek, G.; So, R., Long-term exposure to air pollution and risk of dementia among older individuals of a Danish nationwide administrative cohort. *Environ Int* **2025,** 202, 109607.
9. Wu, Y. T.; Beevers, S.; Barratt, B.; Brayne, C.; Cerin, E.; Franklin, R.; Houlden, V.; Woods, B.; Zied Abozied, E.; Prina, M.; Matthews, F., The longitudinal relationships between the built and natural environment, air pollution, noise and dementia: results from two UK-based cohort studies. *Prev Med* **2025,** 198, 108348.
10. Lefèvre-Arbogast, S.; Scala, P.; Zorilla, A.; Helmer, C.; Chaker, J.; David, A.; Mercier, F.; Samieri, C., Associations of plasma persistent organic pollutants with brain atrophy, cognitive decline and risk of dementia in older adults. *Environ Int* **2025,** 203, 109756.
11. Lefèvre-Arbogast, S.; Duquenne, P.; Helmer, C.; Auriacombe, S.; Sirot, V.; Samieri, C., Association between dietary exposure to chemical contaminants and risk of dementia in older persons. *Environment International* **2024,** 192, 109033.
12. Li, K.; Wu, J.; Mei, Y.; Zhao, J.; Zhou, Q.; Li, Y.; Yang, M.; Xu, J.; Zhao, M.; Xu, Q., Metallomics analysis of metal exposure and cognitive function in older adults: A combined epidemiological and bioinformatics study. *Chemosphere* **2023,** 341, 140049.
13. Fu, Z.; Xu, X.; Cao, L.; Xiang, Q.; Gao, Q.; Duan, H.; Wang, S.; Zhou, L.; Yang, X., Single and joint exposure of Pb, Cd, Hg, Se, Cu, and Zn were associated with cognitive function of older adults. *Sci Rep* **2024,** 14, (1), 28567.
14. Liu, P.; Wang, J.; Mei, P.; Li, J.; Xu, B.; Ren, X.; Chen, X.; Wu, D.; Zhu, F.; Yang, X.; He, M.; Liu, J.; Huang, H., The interaction effect of metals exposure and dietary habit on cognitive function in Chinese older adult cohort. *The journal of nutrition, health & aging* **2024,** 28, (7), 100284.
15. Zhou, Y.; Wang, Y.; Qiu, C.; Man, Y.; Zhu, X.; Tan, S.; Zeng, H.; Guo, X.; Zhang, Z., Associations among blood heavy metals, neurofilament light chains and cognition function in US adults: NHANES 2013-2014. *Ecotoxicology and Environmental Safety* **2025,** 299(no pagination), (118369).
16. Zou, X.; Shi, Y.; Su, J.; Ye, Q.; Lin, F.; Cai, G., Association between 2,4-dichlorophenoxyacetic acid and cognitive impairment in older adults: a cross-sectional study from NHANES 2001-2002 and 2011-2014. *Journal of exposure science & environmental epidemiology* **2024,** 34, (2), 308-316.
17. Ruiz-González, C.; Román, P.; Rueda-Ruzafa, L.; Cardona, D.; Requena, M.; Alarcón, R., Environmental pesticide exposure and Alzheimer's disease in southern Spain: A cross-sectional study. *Psychiatry research* **2024,** 337, 115932.
18. Canovas, R.; Doecke, J. D.; Fowler, C. J.; Fripp, J.; Wang, X.; Mueller, J. F.; Jayarathne, A.; He, C.; Elagali, A.; Dunlop, S.; Masters, C. L., Association of plastic-associated chemicals with Alzheimer's disease amyloid-β and cognitive status. *Journal of Alzheimer's disease : JAD* **2025,** 107, (3), 986-993.
19. Du, H.; Wang, Q.; Chen, Y.; Wu, X.; Jiang, J.; Zhao, Y., Association between mixed exposure of phenols, parabens, phthalates and cognitive function in US elders. *Journal of affective disorders* **2025,** 382, 139-147.

20. Pan, X.; Pollitt, K. J. G.; Liu, S.; Armstrong, F.; Amin, B., Association of per- and polyfluoroalkyl substances (PFAS) exposure with cognitive function in firefighters. *Journal of exposure science & environmental epidemiology* **2026,** 36, (4), 656-666.
21. Wells, G. A., Shea, B., O'Connell, D., Peterson, J., Welch, V., et al. The Newcastle-Ottawa Scale (NOS) for assessing the quality of nonrandomized studies in meta-analysis. http://www.ohri.ca/programs/clinical_epidemiology/oxford.asp
22. Carra, M. C.; Romandini, P.; Romandini, M., Risk of Bias Evaluation of Cross-Sectional Studies: Adaptation of the Newcastle-Ottawa Scale. *J Periodontal Res* **2025**.
